# Graduates of the University of Pennsylvania*Catalogue of the Medical Graduates of the University of Pennsylvania, with a historical sketch of the origin, progress and present state of the Medical Department. Published by the Medical Faculty—1836–7.

**Published:** 1837

**Authors:** 


### II.—ANALYTICAL.

### Art. I.—Medical Graduates of the University of Pennsylvania.*

We have recently had the pleasure of receiving a catalogue of the graduates of our *Alma Mater*. The number is not added up, but they amount to about 3,300. A few of them, in the infancy of the institution, were graduated as bachelors; a practice which has been wisely discontinued since the year 1791.

In examining this register of the dead and the living for seventy years, several reflections are suggested.

1st. If we divide the whole period into two equal terms of thirty-five years, we find that the proportion of inaugural theses written in the Latin language, was far greater in the former than the latter period. Thus the whole number of graduates in one, was 186, nine of whom wrote in that language—the whole number in the other, was 3,114, but seven of whom wrote Latin—about one out of 21 in the former, and one out of 445 in the latter. It would appear, then, that there is a *diminishing* regard for classical learning among our students; a declension which we sincerely regret.

2d. In former times, when graduates were compelled to publish their theses, a greater proportion of them undertook experimental inquiries, than since the University dispensed with that requisition. Thus the repeal of the academic law, was the abolition of one motive to deep study and original research.

3d. It is worthy of note, that almost one-third—that is, 976 out of 3,300—of the graduates are from Virginia: Pennsylva-

* Catalogue of the Medical Graduates of the University of Pennsylvania, with a historical sketch of the origin, progress and present state of the Medical Department. Published by the Medical Faculty—1836-7.

nia stands next in rank, furnishing 776, or nearly a fourth. The two make up more than half of all who have graduated.

4th. If we estimate one physician for every thousand inhabitants, and take the population of the United States at 15,000,000, we have 15,000 practitioners, nearly five times as many as have been graduated in the University of Pennsylvania from its commencement. Let us suppose that twice the number have been graduated in all the other schools of the Union, and still we have had from the *beginning*, but three-fifths of the number now actually employed. It follows, then, that a vast majority of our practitioners have been, and still are, *technically* imperfect in their professional education. Beyond a doubt, many of them have become useful and even distinguished practitioners; but had their education been complete, their success would have been still greater. In this matter we differ very widely and unfortunately, from those kingdoms of Europe, in which no student is allowed to practice till he obtains a diploma.

5th. In looking over the catalogue we observe that a large proportion of those who have founded, or been called to other schools, have been *alumni* of the University of Pennsylvania, which may be emphatically styled *alma mater magna*—a mundane CYBELE—may her *Corybantes* never lose sight of their high responsibilities.

Appended to the catalogue, is a historical sketch of the institution, which will be read with interest by every graduate. Two schools originally existed in Philadelphia, the "College of Philadelphia" and the "University of Pennsylvania." It was not till the year 1791, that they was combined under the title of the latter. The first united Faculty was composed of the following persons:

| Professorships, | Professors. |
|---|---|
| 1. Anatomy, Surgery, and Midwifery, | DR. WILLIAM SHIPPEN, and DR. CASPAR WISTAR, Adjunct; |
| 2. Theory and Practice of Medicine, | DR. ADAM KUHN; |
| 3. Institutes of Medicine, and Clinical Medicine, | DR. BENJAMIN RUSH; |
| 4. Chemistry, | DR. JAMES HUTCHINSON; |
| 5. Materia Medica, | DR. SAMUEL P. GRIFFITHS; |
| 6. Botany and Natural History, | DR. BENJAMIN S. BARTON. |

We have only room to enumerate those who subsequently held appointments, or are now in office:—Dr. JOHN CARSON, who died before he officiated; Dr. Philip Syng Physick, Dr. James Woodhouse, Dr. Nathaniel Chapman, Dr. John Redman Coxe, Dr. John Syng Dorsey, Dr. Thomas C. James, Dr. Robert Hare, Dr. William Gibson, Dr. William E. Horner, Dr. William P. Dewees, Dr. Samuel Jackson, Dr. George B. Wood, Dr. Hugh L. Hodge.

We shall conclude this notice with two or three *reflections* on the institution, which, we hope, will not be thought presumptuous or irreverent, although made by a son residing in the *Backwoods.*

The Medical Department of the University of Pennsylvania, has done much for the medical profession of the New World; she is deservedly respected in the Old; and, notwithstanding the constant rise of other schools, maintains herself, it appears, at the average number of 400 pupils. Still, it must be admitted, that her long continued prosperity is not entirely the result of her organization, or the manner in which *all* her chairs have been filled. Her policy has been clearly that of *conservation.* She has, at all times, been cautious. The moral power, which early and continued comparative superiority placed in her hands, has not been put forth upon the profession, with as much enterprise as might have been expected. The original error (admitted to us twenty years ago by one of her ablest professors) of limiting her session to four months, has never been corrected, as it might have been —as she might now correct it with a stroke of the pen, and see her example followed throughout the Union. Her present scope and arrangement of the branches of medical science is essentially the same with that of 1791. She has not with freedom and facility, adapted herself to the improvements of the last forty years. When her ample halls were more thronged than they had ever been before, she so far forgot herself as to oppose the establishment, in Philadelphia, of another school by her own graduates. Her precepts have never been rash, but not always wise and exciting. Finally, her career has presented little of the positive to condemn, but less to admire than could have been wished. To expect that any intimations from this distant point, will modify her policy, or animate her efforts, would savor of vanity; and still we cannot forget, that the passiveness of a parent has sometimes been overcome by the impulsive suggestions of the child; and that hints from the humble, have occasionally exerted a salutary influence on the most exalted.

In conclusion, we would notify to all our fellow graduates, that the Faculty are desirous of putting each of them in possession of a catalogue, and that whoever sends his name and address to the Dean, will be supplied. D.

